# Treatment Outcomes of Pediatric Acute Myeloid Leukemia in the Yeungnam Region: A Multicenter Retrospective Study of the Study Alliance of Yeungnam Pediatric Hematology–Oncology (SAYPH)

**DOI:** 10.3390/children8020109

**Published:** 2021-02-05

**Authors:** Jae Min Lee, Eu Jeen Yang, Kyung Mi Park, Young-Ho Lee, Heewon Chueh, Jeong Ok Hah, Ji Kyoung Park, Jae Young Lim, Eun Sil Park, Sang Kyu Park, Heung Sik Kim, Ye Jee Shim, Jeong A. Park, Eun Jin Choi, Kun Soo Lee, Ji Yoon Kim, Young Tak Lim

**Affiliations:** 1 Department of Pediatrics, College of Medicine, Yeungnam University, Daegu 42415, Korea; mopic@yu.ac.kr; 2 Department of Pediatrics, Pusan National University Children’s Hospital, Pusan National University, School of Medicine, Yangsan 50612, Korea; 41sirius@hanmail.net (E.J.Y.); mayani@hanmail.net (K.M.P.); 3 Department of Pediatrics, Hanyang University College of Medicine, Hanyang University Medical Center, Seoul 04763, Korea; cord@hanyang.ac.kr; 4 Department of Pediatrics, Dong-A University College of Medicine, Busan 49201, Korea; caaf80@empal.com; 5 Department of Pediatrics, Daegu Fatima Hospital, Daegu 41199, Korea; johah@med.yu.ac.kr; 6 Department of Pediatrics, Inje University College of Medicine, Busan Paik Hospital, Busan 47392, Korea; pjk4285@hanmail.net; 7 Department of Pediatrics, Gyeongsang National University College of Medicine, Jinju 52727, Korea; pedneu@gnu.ac.kr (J.Y.L.); espark@gnu.ac.kr (E.S.P.); 8 Department of Pediatrics, Ulsan University Hospital, Ulsan 44033, Korea; sangulsan@hanmail.net; 9 Department of Pediatrics, Keimyung University School of Medicine, Keimyung University Daegu Dongsan Hospital, Daegu 41931, Korea; kimhs@dsmc.or.kr; 10 Department of Pediatrics, Keimyung University School of Medicine, Keimyung University Dongsan Hospital, Daegu 42601, Korea; yejeeshim@dsmc.or.kr; 11 Department of Pediatrics, Inje University Haeundae Paik Hospital, Busan 41808, Korea; jeonga95@gmail.com; 12 Department of Pediatrics, Memorial Sloan Kettering Cancer Center, Manhattan, NY 10065, USA; 13 Department of Pediatrics, Daegu Catholic University Medical Center, Daegu 42472, Korea; ejchoi2@cu.ac.kr; 14 Department of Pediatrics, School of Medicine, Kyungpook National University, Daegu 41944, Korea; kslee@knu.ac.kr (K.S.L.); phojyk@gmail.com (J.Y.K.)

**Keywords:** acute myeloid leukemia, hematopoietic stem cell transplantation, prognosis, pediatric, childhood, Korea

## Abstract

Acute myeloid leukemia (AML) is the second most common pediatric leukemia, with a survival rate of 70%. In this retrospective study, we evaluated the treatment outcomes of pediatric AML among 144 patients diagnosed between 2000 and 2013. After induction, 80.6% of patients achieved complete remission (CR). The 5-year overall survival (OS) and event-free survival (EFS) rates were 58.8 ± 4.2% and 49.8 ± 4.2%, respectively. Based on the response to induction therapy, the 5-year OS was 66.9 ± 5.7% in patients with CR (*p* < 0.001). Ninety-nine patients with CR after induction therapy were examined, and their 5-year OS and EFS were 66.4 ± 4.9% and 56.3 ± 5.1%, respectively. The 5-year OS rates according to treatment were 59.9 ± 7.4% in the chemotherapy group and 72.3 ± 6.3% in the hematopoietic stem cell transplantation (HSCT) group (*p* = 0.089). The EFS was 50.1 ± 7.4% in the chemotherapy group and 61.7 ± 6.9% in the HSCT group (*p* = 0.098). OS and EFS according to cytogenetics were insignificant. Our findings confirmed that the response to induction treatment was important for survival and HSCT had no significant survival benefits compared with those of chemotherapy. Moreover, many early induction deaths under the age of 2 years were observed.

## 1. Introduction

Pediatric acute myeloid leukemia (AML) accounts for approximately 25% of all childhood leukemias in Korea and is the second most common leukemia type after acute lymphocytic leukemia in the country. The age-standardized rate of childhood acute myeloid leukemia in Korea for the period 1999–2011 is 28.3 per million [1]. The prognoses of children with AML were previously poor; however, there has been an improvement over the past 30 years [2,3]. The survival rate associated with childhood AML has greatly improved owing to the development of risk stratification, advances in treatment strategies such as a combination of intensive myelosuppressive chemotherapy and hematopoietic stem cell transplantation (HSCT), and the provision of supportive care in particular [2]. As a result, several groups now demonstrate complete remission (CR) rates of 85–95%, relapse rates of 20–30%, event-free survival (EFS) rates of 50–60%, and overall survival (OS) rates of 70–80% [4,5]. The increase in the level of awareness related to leukemogenic genetic events that define the subsets of AML has led to the development of novel strategies to inhibit the underlying events contributing to leukemogenesis. However, for the vast majority of mutations, it is not sufficient to assume that target expression will correspond to a response to targeted therapy [6].

Therefore, in the present study, we reviewed the treatment experience of pediatric patients with AML across 10 tertiary medical institutions in South Korea to identify the characteristics and outcomes of the disease and use the results as the basis for risk stratification and future prospective studies.

## 2. Materials and Methods

### 2.1. Data Collection

**Figure 1:**
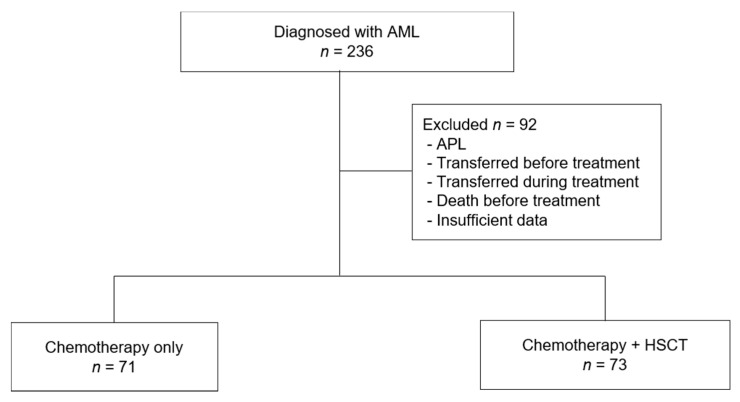
Flowchart of patient enrollment. AML, acute myeloid leukemia; APL, acute promyelocytic leukemia; HSCT, hematopoietic stem cell transplantation.

We performed a retrospective study among patients with AML diagnosed between 2000 and 2013. In all, 236 patients aged <20 years at the time of diagnosis were enrolled from 10 tertiary medical centers in the Yeungnam region in Korea. Among them, 26 patients with acute promyelocytic leukemia were excluded, 36 were transferred to medical centers outside the region before initiation of treatment, 2 died before the commencement of treatment, 3 had insufficient data in their medical records, and 25 were transferred during treatment. Therefore, 144 patients were included in the final analysis of treatment outcomes (Figure 1).

### 2.2. Definition

AML diagnosis was confirmed when the number of leukemia cells in the bone marrow (BM) aspirate was ≥20%, and relapse was defined as a blast count >5% in the BM. Cytogenetic analyses were performed on metaphases from BM aspirates obtained at diagnosis at the regional laboratories as part of routine care. Specific groups according to the World Health Organization (WHO) classification, based on the presence of abnormalities, such as core-binding factor leukemias t(8;21)(q22;q22) and inv(16)(p13.1q22) or t(16;16)(p13.1;q22); t(9;11)(p21;q23); mixed lineage leukemia (MLL) translocations with other partner genes, inv(3)(q21q26.2), or t(3;3)(q21;q26.2); and monosomy seven, were defined irrespective of the presence of other aberrations. A normal karyotype was defined as a karyotype with the absence of clonal cytogenetic aberrations and FISH negativity for t(8;21), inv(16), and MLL aberrations. A complex karyotype was defined as a karyotype with the presence of three or more chromosomal abnormalities in the absence of one of the WHO-designated recurring translocations or inversions [7]. Other cytogenetic abnormalities were defined as the presence of clonal aberrations other than those classified above.

The cytogenetic risk status of the patients was stratified according to the AML committee of the international BFM study group criteria [8]. The central nervous system (CNS) status was based on the cell contents in the cerebrospinal fluid (CSF): CNS1, nontraumatic puncture without leukemic blasts; CNS2, blasts detected using cytocentrifugation of the CSF with <5 nucleated cells/mm^3^; and CNS3, overt CNS leukemia as per the Rome Workshop criteria (at least 5 nucleated cells/mm^3^ with identified blasts, or the presence of cranial nerve palsies). Traumatic lumbar puncture was defined as the presence of >10 erythrocytes/mm^3^ in the CSF or macroscopically contaminated CSF.

The definitions of response after induction were as follows: CR, BM with a blast count <5%, absence of peripheral (PB) leukemia cells, absence of extramedullary disease; partial response (PR), BM with 5–15% blast cells, absence of PB leukemia cells, and evidence of normal hematopoietic cell regeneration. No response (NR) was confirmed when a patient did not achieve CR or PR and survived beyond the first 6 weeks of treatment. Early induction death was defined as death occurring within the first 6 weeks of treatment. 

OS was defined as the time from diagnosis to death or with living patients censored at last follow-up. EFS was calculated from the date of diagnosis to the last follow-up or first event (failure to achieve remission, relapse, second malignancy, or death due to any cause, whichever occurred first).

### 2.3. Treatment

After being diagnosed with AML, the patients received induction and consolidation chemotherapy according to each institution’s policy. AML 2000, AML-BFM 2004, DCTER, 7 + 3, and 5 + 3 regimens were used for induction chemotherapy, whereas consolidation chemotherapy was performed using different regimens. Maintenance chemotherapy or HSCT was performed depending on the associated risk, donor availability, and institutional policy.

### 2.4. Statistical Analysis

The patients’ demographic, clinical, and pathological data were compared among the three response groups using the Kruskal-Wallis test for continuous variables and chi-square test for categorical variables. EFS and OS values, along with their standard errors, were estimated using the Kaplan-Meier method. The log-rank test was used to compare the survival rates. When comparing the survival outcomes between chemotherapy and HSCT, patients with secondary AML and unknown cytogenetic data were excluded while patients with CR response to induction treatment were included in the study. SPSS ver. 25.0 (IBM Inc., Armonk, NY, USA) was used for all statistical analyses. All *p*-values were two-sided, and *p* < 0.05 was considered significant.

### 2.5. Ethics Statement

This multicenter, retrospective study was approved by the Daegu Joint Institutional Review Board (Approval No. 2015-08-002) and performed according to the Declaration of Helsinki 1975.

## 3. Results

### 3.1. Patient Characteristics

The characteristics of the patients are summarized in Table 1. The median age at diagnosis was 9.1 (0.0–19.5) years, and the median white blood cell (WBC) count at diagnosis was 15,300/μL (630–319,700). WBC counts <20,000/μL were observed in 79 (54.9%) patients. The median follow-up duration was 9.4 (range, 0.4–18.5) years. Male predominance was observed (female: male = 1:1.82) (Table 1). Regarding the French–American–British (FAB) classification, M2 was the most prevalent (42.4%) subtype. Most patients showed de novo AML (96.5%), and in five patients, the disease was secondary to either a myelodysplastic syndrome or previous therapy for another malignancy (secondary AML). There were seven (4.9%) patients with Down syndrome. CNS1 was observed in 114 (79.2%) patients, and six (4.2%) patients experienced a traumatic tap at the time of diagnosis. Twelve (8.3%) patients had chloroma at the time of diagnosis.

### 3.2. Cytogenetics Studies

**Figure 2:**
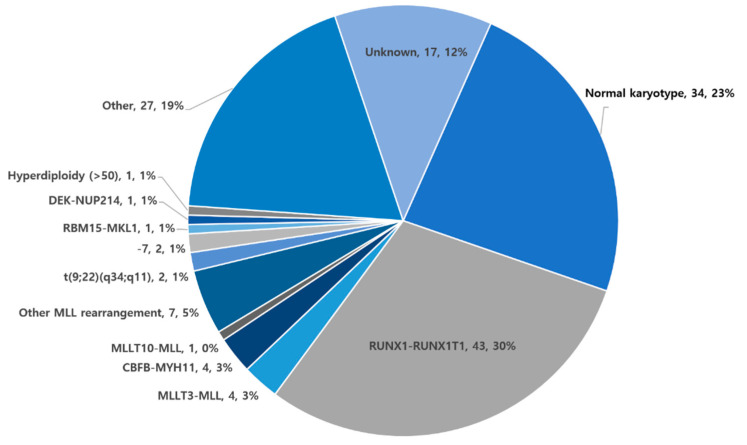
Cytogenetics of patients diagnosed with acute myeloid leukemia. Expressed as cytogenetic anomality, number of patients, %.

Cytogenetic data were available for 127 (88.1%) patients. The most frequently observed chromosomal abnormality in this study was the RUNX1-RUNX1T1 fusion (*n* = 43, 30%). MLL rearrangements (MLL-MLLT10, MLLT3-MLL, and other MLL rearrangements) were found in 12 (8.3%) patients. Thirty-four (23%) patients were cytogenetically normal. (Figure 2). Regarding cytogenetic risk classification, 41 (29.1%) patients were assigned to the favorable group, 69 (48.9%) to the intermediate group, 15 (10.6%) to the adverse group, and 16 (11.3%) to the unknown group (Table 1).

### 3.3. Treatment

The regimen used for induction was 84 (58.3%) DCTER, 12 7 + 3 regimen, and 48 (33.3%) other. The median number of cycles of induction and consolidation was two (range 1–4) and three (range 1–8), respectively. After induction chemotherapy, 116 (80.6%) patients achieved CR, 9 (6.3%) achieved PR, and 6 (4.2%) showed NR. Eleven (7.6%) early induction deaths were observed. Treatment response after induction chemotherapy according to the age group was as follows: in the 0–1.99-year group, 13 patients (59.1%) showed CR, 4 (18.2%) showed PR, and 4 (18.2%) had early deaths. The CR rates in the 2–9.99-year and ≥10-year groups were 46 (83.6%) and 57 (85.1%), respectively, and early induction deaths were observed in five (9.1%) and two (3.0%) patients, respectively (Table 2). The 0–1.99-year group showed a lower CR rate and higher early induction death rate than the ≥2-year group (*p* = 0.021). According to cytogenetics, 90.2% showed CR with favorable cytogenetics, 76.1% showed CR with intermediate cytogenetics, and 73.3% showed CR with adverse cytogenetics (*p* = 0.911).

### 3.4. Outcomes

**Figure 3:**
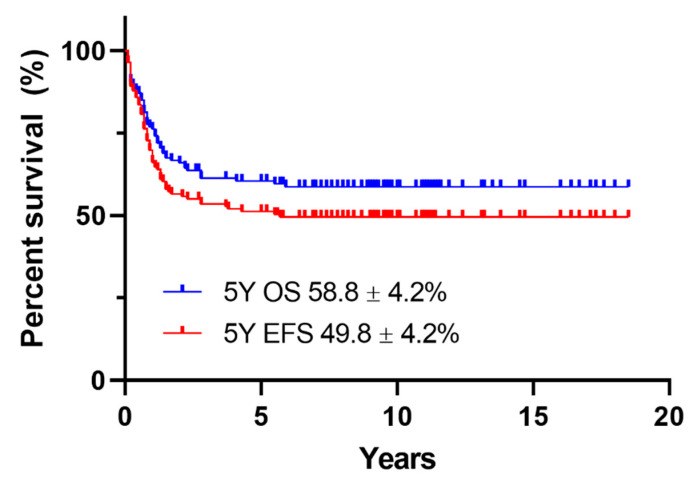
The 5-year OS and EFS rates of patients. OS, overall survival; EFS, event-free survival.

**Figure 4:**
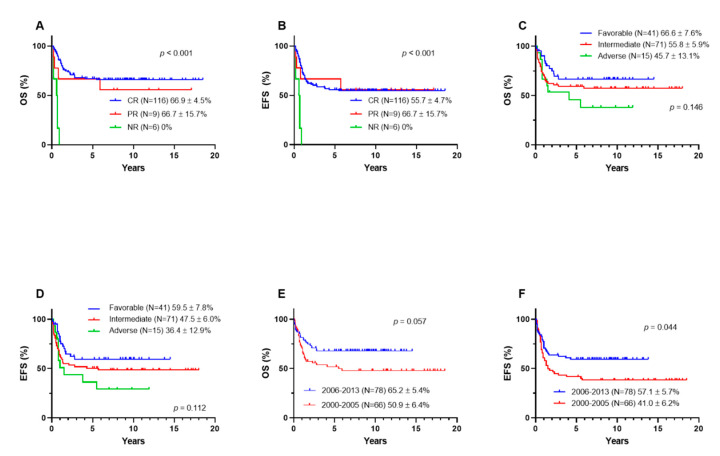
Treatment outcome of study patients. (**A**) OS and (**B**) EFS, according to induction response, (**C**) OS and (**D**) EFS, according to cytogenetics, (**E**) OS and (**F**) EFS, according to period. CR, complete response; PR, partial response; NR, no response; OS, overall survival; EFS, event-free survival.

The 5-year OS and EFS rates were 58.8 ± 4.2% and 49.8 ± 4.2%, respectively (Figure 3). The 5-year OS rates, according to the response to induction therapy, were 66.9 ± 4.5% in the CR group, 66.7 ± 15.7% in the PR group, and 0% in the NR group (Figure 4A). The 5-year EFS rates, according to the response to induction therapy, were 55.7 ± 4.7% in the CR group, 66.7 ± 15.7% in the PR group, and 0% in the NR group (Figure 4B). The 5-year OS rates, according to cytogenetic data, were 66.6 ± 7.6% in the favorable group, 55.8 ± 5.9% in the intermediate group, and 45.7 ± 13.1% in the adverse group (*p* = 0.146) (Figure 4C). The 5-year EFS rates, according to cytogenetic data, were 59.5 ± 7.8% in the favorable group, 47 ± 6.0% in the intermediate group, and 36.4 ± 12.9% in the adverse group (*p* = 0.112) (Figure 4D). The 5-year EFS rate significantly improved after 2006; however, the OS rate did not improve (OS 65.2 ± 5.4% vs. 50.9 ± 6.4%, *p* = 0.057; EFS 57.1 ± 5.7% vs. 41.0 ± 6.2%, *p* = 0.044) (Figure 4E,F). In seven patients with Down syndrome, one patient underwent induction chemotherapy with DCTER and one patient underwent the 7 + 3 regimen, and none of them received transplantation. Four out of seven survived.

### 3.5. Risk Factors

In the univariate analysis, age, sex, the WBC count at diagnosis, the FAB subtype, the cytogenetic group, extramedullary involvement, and the CNS status did not affect the treatment outcomes. Regarding the 5-year OS rate according to the type of AML, patients with secondary AML showed worse outcomes than patients with de novo AML (60.2 ± 4.2% vs. 20.0 ± 17.9%, *p* = 0.041) (Table 3).

### 3.6. Hematopoietic Stem Cell Transplantation vs. Chemotherapy

Excluding patients with secondary AML and unknown cytogenetic data, 99 patients with CR1 after induction were examined, and the 5-year OS and EFS rates were 66.4 ± 4.9% and 56.3 ± 5.1%, respectively (Table 4). The 5-year OS rates according to treatment were 59.9 ± 7.4% in the chemotherapy group and 72.3 ± 6.3% in the transplantation group (*p* = 0.089). The EFS rates according to treatment were 50.1 ± 7.4% in the chemotherapy group and 61.7 ± 6.9% in the transplantation group (*p* = 0.098). OS and EFS rates according to cytogenetic data were insignificant.

## 4. Discussion

The present study evaluated the survival outcomes of patients diagnosed with pediatric AML in the Yeungnam region of Korea. In this retrospective study, we evaluated the treatment outcomes of pediatric AML among 144 patients diagnosed between 2000 and 2013. Of all patients, 80.6% achieved CR after induction. The 5-year OS and EFS rates were 58.8 ± 4.2% and 49.8 ± 4.2%, respectively. Ninety-nine patients with CR after induction therapy were examined, and the 5-year OS rates according to treatment were 59.9 ± 7.4% in the chemotherapy group and 72.3 ± 6.3% in the HSCT group (*p* = 0.089). The EFS was 50.1 ± 7.4% in the chemotherapy group and 61.7 ± 6.9% in the HSCT group (*p* = 0.098). OS and EFS according to cytogenetics were insignificant.

Pediatric AML accounts for 15–20% of all pediatric acute leukemias. The associated survival rates have increased to 70% over the past few decades owing to improvements in supportive care, optimized risk stratification, and intensified chemotherapy [2,4,5,9]. Treatment for the disease includes a combination of intensive anthracycline- and cytarabine-containing chemotherapy, as well as stem cell transplantation in selective high-risk genetic cases or slow responders.

In the NOPHO 2004 trial, after the first course of chemotherapy, planning for further treatment was based on the observed response. The overall remission rate was 97.4%, and 92% of patients achieved remission after the second course of chemotherapy. The good and poor responder groups showed a high EFS of 61% and 82%, respectively, but the intermediate group showed a low EFS of 35%, suggesting that the intensification of consolidation therapy using HSCT would be required in this group [10].

In our study, 80.6% of patients achieved remission after induction. The OS and EFS rates of patients with CR were 66.4 ± 4.9% and 56.3 ± 5.1%, respectively. However, there was no difference in the survival outcome between chemotherapy and HSCT in these patients.

In a multicenter Dutch–Belgian study (DB AML-01), patients achieving CR after two induction courses continued with three consolidation courses without HSCT in CR1. The 3-year EFS and OS rates were 52.6% and 74%, respectively. It was concluded that DB AML-01 response-guided therapy showed favorable OS without HSCT [11]. In the AIEOP AML 2002/01 trial, 87% achieved CR and the 8-year OS and EFS rates were 68% and 55%, respectively [12,13]. The treatment was stratified according to the risk group, where patients with core-binding factor leukemia achieving CR after the first induction course was considered as the standard risk, whereas the others were assigned to the high-risk group. The findings revealed that the risk group, WBC > 100 × 109/L at diagnosis, and monosomal karyotype predicted poor EFS. In our study, the 5-year OS and EFS rates were 58.8% and 49.8%, respectively, wherein response to induction treatment was a predictor of survival outcome.

Application of HSCT must be carefully considered for potential benefits. Our study demonstrated that there were no differences in the OS and EFS between patients who received chemotherapy and patients who underwent HSCT as post-remission consolidation therapy. According to the cytogenetics data, the survival outcome of chemotherapy and HSCT did not show any significant difference. In addition, in a previous study, we had reported that there is no difference in the survival outcome based on donor type and stem cell source after allogenic HSCT [14]. 

In a recent study of pediatric AML patients in China, unmanipulated haploidentical HSCT may overcome the poor prognostic significance of resistant to the first course of induction chemotherapy in children with AML [15]. Another study reported that haploidentical HSCT showed lower relapse incidence and better EFS in patients with intermediate-risk AML in first CR compared to the chemotherapy group. In the future, the study of haploidentical HSCT in the first CR of pediatric AML patients may be promising [16].

In this study, among adverse cytogenetics, OS and EFS in the transplantation group showed a tendency that the treatment outcome was better than that of chemotherapy (*p* = 0.074). Although it is not statistically significant, this seems to be due to the small number of patients involved with adverse cytogenetics. In adverse cytogenetics, transplantation may help improve survival, and if there is no suitable donor, haploidentical HSCT should also be considered. In addition, HSCT should be considered in secondary AML and AML with chloroma, which showed relatively poor survival.

Chloroma, also known as myeloid sarcoma (MS), is a malignant extramedullary tumor that involves immature cells of myeloid origin. The reported incidence of MS in pediatric AML is 10–20% [17,18]. It may occur de novo or concurrently, or precede the diagnosis of AML, myelodysplastic syndrome, or chronic myeloid leukemia. MS can also be a manifestation of disease relapse. Usually, the presence of MS is associated with poor prognoses [19].

One study on COG reported that the prognosis was worse in patients with extramedullary leukemia with skin involvement than in patients with non-skin involvement, and CNS positivity was more common in this group. Patients with non-skin involvement reported a high incidence of t(8; 21)(q22; q22) abnormalities and a good prognosis. Local radiotherapy did not improve the survival outcome in these patients [20]. A single center study by Zhou et al. reported that MS is as a poor prognostic factor in AML with non-favorable cytogenetics but not in AML with favorable cytogenetics [18]. In our study, chloroma was detected in 12 (8.3%) patients at diagnosis, a number lower than that reported in previous studies, and the EFS values in these patients were low. In our study, owing to the small number of patients, prognostic factors in patients with chloroma could not be analyzed, and more patients will need to be examined to determine the prognosis associated with MS.

In the UK Medical Research Council Acute Myeloid Leukemia 12 (UK MCR AML 12) trial, patients with AML were randomized to receive mitoxantrone/cytarabine/etoposide or daunorubicin/cytarabine/etoposide as induction chemotherapy, and 270 patients entered the second randomization phase, receiving four or five treatment courses in total. There was no difference in the CR rate between the induction regimens, but there was a benefit associated with mitoxantrone with regard to the relapse rate. However, this did not translate to better EFS or OS rates. The results of the second randomization did not show a survival benefit in association with the fifth course of treatment, suggesting a ceiling of benefit for conventional chemotherapy and demonstrating the need for new agents. In that study, the EFS was superior to that observed in the preceding AML10 trial, partly owing to fewer deaths in remission, highlighting the importance of supportive care [21].

In our study, the 5-year OS and EFS rates were 58.8 ± 4.2% and 49.8 ± 4.2%, respectively. However, when the treatment period was analyzed, it was confirmed that the EFS associated with AML had shown improvement since 2006 (5-year EFS 57.1 ± 5.7% vs. 41.0 ± 6.2%), without major changes in the protocol. This may be because the level of supportive care has improved in recent years, as noted in the UK MRC AML12 trial. Owing to the retrospective nature of this study, an accurate treatment-related mortality analysis was not possible; hence, this study has limitations in establishing the role of supportive care in improved EFS.

In a Nordic study, cytogenetics and the presented WBC count were the only independent prognostic factors associated with overall survival; age was not an independent prognostic factor [22]. In our study, age, sex, the WBC count at diagnosis, the FAB subtype, cytogenetics, and extramedullary or CNS involvement were found to be independent prognostic factors associated with survival. Only the induction response was a predictor of survival outcome.

According to our findings, four (18.2%) of the 22 children aged <2 years died. The rate of early death during induction was higher than that in other age groups. Therefore, it is necessary to develop a treatment protocol to increase the rate of CR while reducing the levels of toxicity in this patient group.

The results of the present multicenter study conducted in the Yeungnam region, Korea, are very meaningful as they reflect the therapeutic results of pediatric AML, when patients are treated with various protocols in a real-world setting. However, owing to the limitations associated with retrospective studies, the protocols were not uniform across the institutions. Some data about the cause of death were not available. Additionally, the results of genetic tests were not available for some patients.

## 5. Conclusions

Our findings confirmed that the response to induction treatment was important for survival, HSCT had no significant survival benefits compared with those of chemotherapy, and there were many early induction deaths under the age of 2 years.

Further prospective studies must focus on the development of new treatments aimed at reducing the rate of toxicity and improving the remission rate in patients aged <2 years. Additionally, it is crucial to develop novel therapies with improved long-term outcomes; moreover, careful identification of high-risk patients who can benefit from HSCT is required.

## Figures and Tables

**Table 1:** Baseline characteristics of patients.

		All periods		2000–2006		2006–2013		
		*n*	(%)	*n*	(%)	*n*	(%)	*p*
No. of Patients		144		66		78		
Gender								0.406
	Male	93	(64.6)	45	(68.2)	48	(61.5)	
	Female	51	(35.4)	21	(31.8)	30	(38.5)	
Age								0.876
	≤1.99	22	(15.3)	9	(13.6)	13	(16.7)	
	2–9.99	55	(38.2)	26	(39.4)	29	(37.2)	
	>10	67	(46.5)	31	(47.0)	36	(46.2)	
WBC								0.941
	≤19999	79	(54.9)	37	(56.1)	42	(53.8)	
	20000–99999	53	(36.8)	24	(36.4)	29	(37.2)	
	>100000	12	(8.3)	5	(7.6)	7	(9.0)	
FAB subtype								0.579
	M0	3	(2.1)	2	(3.0)	1	(1.3)	
	M1	28	(19.4)	14	(21.2)	14	(17.9)	
	M2	61	(42.4)	29	(43.9)	32	(41.0)	
	M4	13	(9.0)	6	(9.1)	7	(9.0)	
	M5	12	(8.3)	6	(9.1)	6	(7.7)	
	M6	2	(1.4)	0	0.0	2	(2.6)	
	M7	10	(6.9)	2	(3.0)	8	(10.3)	
	Unclassified	15	(10.4)	7	(10.6)	8	(10.3)	
Cytogenetics								0.115
	Favorable	41	(29.1)	14	(22.2)	27	(34.6)	
	Intermediate	71	(49.3)	32	(48.5)	39	(50.0)	
	Adverse	15	(10.6)	9	(14.3)	6	(7.7)	
	Unknown	17	(11.8)	11	(16.7)	6	(7.7)	
Type								0.79
	De novo AML	139	(96.5)	64	(97.0)	75	(96.2)	
	Secondary AML	5	(3.5)	2	(3.0)	3	(3.8)	
CNS								0.023
	CNS1	114	(79.2)	47	(71.2)	67	(85.9)	
	CNS2	1	(7.0)	0	0.0	1	(1.3)	
	CNS3	0	0.0	0	0.0	0	0.0	
	Traumatic tap	6	(4.2)	2	(3.0)	4	(5.1)	
	Unknown, not done	23	(16.0)	17	(25.8)	6	(7.7)	
Extramedullary								0.13
	None	132	(91.7)	58	(87.9)	74	(94.9)	
	Chloroma	12	(8.3)	8	(12.1)	4	(5.1)	
Treatment								0.411
	Chemotherapy	71	(49.3)	35	(53.0)	36	(46.2)	
	HSCT	73	(50.7)	31	(47.0)	42	(53.8)	

AML, acute myeloid leukemia; CNS, central nervous system; FAB, French–American–British classification; WBC, white blood cell; HSCT, hematopoietic stem cell transplantation.

**Table 2:** Treatment response after induction chemotherapy by age and cytogenetics.

			N (%)		CR		PR		NR		Early Death during Induction		Unknown		*p* ^a^	*p* ^b^
Age (year)																
	Total		144 (100)		116 (80.6)		9 (6.3)		6 (4.2)		11 (7.6)		2 (1.4)		0.058	0.021
	≤1.99		22 (15.3)		13 (59.1)		4 (18.2)		1 (4.5)		4 (18.2)		0 (0)			
	≥2		122 (84.7)		103 (84.4)		5 (4.1)		5 (4.1)		7 (5.7)		2 (1.6)			
		2–9.99		55 (38.2)		46 (83.6)		2 (3.6)		2 (3.6)		5 (9.1)		0 (0)		
		≥10		67 (46.5)		57 (85.1)		3 (4.5)		3 (4.5)		2 (3.0)		2 (3.0)		
Cytogenetics																
	Favorable		41 (28.5)		37 (90.2)		0 (0)		2 (4.9)		2 (4.9)		0 (0)			
	Intermediate		71 (49.3)		54 (76.1)		5 (7.0)		4 (5.6)		7 (9.9)		1 (1.4)			
	Adverse		15 (10.4)		11 (73.3)		2 (13.3)		0 (0)		1 (6.7)		1 (6.7)			
	Unknown		17 (11.8)		14 (82.4)		2 (11.8)		0 (0)		1 (5.9)		0 (0)			

CR, complete response; PR, partial response; NR, no response. ^a^ *p*-values calculated for comparison among three groups (0–1.99, 2–9.99, and ≥10 year); ^b^ *p*-values calculated for comparison between two groups (0–1.99 and ≥2 year).

**Table 3:** Univariate analysis of survival.

Risk Factor		No. (%)	5-Year OS	*p*	5-Year EFS	*p*
Age				0.252		0.314
	0–1.99	22 (15.3)	48.0 ± 11.0		45.0 ± 10.7	
	2–9.99	55 (38.2)	67.0 ± 6.4		57.5 ± 6.7	
	≥10	67 (46.5)	55.4 ± 6.2		44.9 ± 6.2	
Sex				0.776		0.925
	Male	93 (64.6)	58.3 ± 5.2		49.2 ± 5.3	
	Female	51 (35.4)	59.8 ± 7.0		50.7 ± 7.0	
WBC				0.339		0.413
	0–19,999	79 (54.9)	63.5 ± 5.5		54.3 ± 5.7	
	20,000–99,999	53 (36.8)	51.8 ± 7.0		45.2 ± 6.8	
	>100,000	12 (8.3)	58.3 ± 14.2		38.9 ± 14.7	
FAB				0.485		0.312
	M0	3 (2.1)	0		0	
	M1	28 (19.4)	51.4 ± 9.8		37.7 ± 9.3	
	M2	61 (42.4)	62.9 ± 6.3		56.7 ± 6.4	
	M4	13 (9.0)	61.5 ± 13.5		38.5 ± 13.5	
	M5	12 (8.3)	50.0 ± 14.4		40.0 ± 14.6	
	M6	2 (1.4)	50.0 ± 35.4		50.0 ± 35.4	
	M7	10 (6.9)	60.0 ± 15.5		60.0 ± 15.5	
	Unclassifiable/unknown	15 (10.4)	66.0 ± 12.4		60.0 ± 12.6	
Genetics				0.238		0.224
	Favorable	41 (28.5)	66.6 ± 7.9		59.5 ± 7.8	
	Intermediate	71 (49.3)	55.8 ± 5.9		47.5 ± 6.0	
	Adverse	15 (10.4)	45.7 ± 13.1		36.4 ± 12.9	
	Unknown	17 (11.8)	64.2 ± 11.8		47.1 ± 12.1	
Type				0.041		0.112
	De novo AML	139 (96.5)	60.2 ± 4.2		50.9 ± 4.3	
	Secondary AML	5 (3.5)	20.0 ± 17.9		20.0 ± 17.9	
Extramedullary				0.153		0.067
	None	132 (91.7)	60.6 ± 4.2		52.1 ± 4.4	
	Chloroma	12 (8.3)	35.0 ± 15.4		25.0± 12.5	
CNS				0.212		0.145
	CNS 1	114 (79.2)	67.7 ± 4.5		59.8 ± 4.6	
	CNS 2	1 (0.7)	100		100	
	Traumatic tap	6 (4.2)	50.0 ± 20.4		33.3 ± 19.2	
	Unknown, not done	23 (16.0)	39.1 ± 10.2		34.8 ± 9.9	
Induction response				<0.001		<0.001
	CR	116 (88.5)	66.9 ± 4.5		55.7 ± 4.7	
	PR	9 (6.9)	66.7 ± 15.7		66.7 ± 15.7	
	NR	6 (4.6)	0		0	

AML, acute myeloid leukemia; CNS, central nervous system; EFS, event-free survival; FAB, French–American–British classification; OS, overall survival; WBC, white blood cell; CR, complete remission; PR, partial response; NR, no response.

**Table 4:** Comparing survival between chemotherapy and transplantation in CR1 according to cytogenetics.

		N	Overall Survival	*p*	Event-Free Survival	*p*
All patients		99	66.4 ± 4.9		56.3 ± 5.1	
Chemotherapy vs. transplantation				0.089		0.098
	Chemotherapy	47	59.9 ± 7.4		50.1 ± 7.4	
	Transplantation	52	72.3 ± 6.3		61.7 ± 6.9	
Favorable cytogenetics				0.657		0.905
	Chemotherapy	19	76.7 ± 10.2		66.7 ± 11.1	
	Transplantation	18	70.9 ± 11.0		65.3 ± 11.6	
Intermediate cytogenetics				0.068		0.140
	Chemotherapy	22	53.3 ± 10.8		45.5 ± 10.6	
	Transplantation	30	73.0 ± 8.2		58.6 ± 9.2	
Adverse cytogenetics				0.138		0.074
	Chemotherapy	6	33.3 ± 19.2		16.7 ± 15.2	
	Transplantation	4	75.0 ± 21.7		66.7 ± 27.2	

Five-year estimates (2 SE) are shown. Patients with secondary AML and unknown cytogenetics are excluded and patients with CR induction response are included. CR1, first complete remission.

## Data Availability

The data are not publicly available due to ethical reasons and patient privacy.

## References

[1] Park H.J., Moon E.K., Yoon J.Y., Oh C.M., Jung K.W., Park B.K., Shin H.Y., Won Y.J. (2016). Incidence and Survival of Childhood Cancer in Korea. Cancer Res. Treat. Off. J. Korean Cancer Assoc..

[2] Zwaan C.M., Kolb E.A., Reinhardt D., Abrahamsson J., Adachi S., Aplenc R., De Bont E.S., De Moerloose B., Dworzak M., Gibson B.E. (2015). Collaborative Efforts Driving Progress in Pediatric Acute Myeloid Leukemia. J. Clin. Oncol. Off. J. Am. Soc. Clin. Oncol..

[3] Jae Wook L., Bin C. (2015). Diagnosis and Treatment of Pediatric Acute Myeloid Leukemia. Clin. Pediatr. Hematol. Oncol..

[4] Rubnitz J.E. (2017). Current Management of Childhood Acute Myeloid Leukemia. Paediatr Drugs.

[5] Taga T., Tomizawa D., Takahashi H., Adachi S. (2016). Acute myeloid leukemia in children: Current status and future directions. Pediatr. Int..

[6] Kolb E.A., Meshinchi S. (2015). Acute myeloid leukemia in children and adolescents: Identification of new molecular targets brings promise of new therapies. Hematology/the Education Program of the American Society of Hematology. American Society of Hematology. Educ. Program.

[7] Arber D.A., Orazi A., Hasserjian R., Thiele J., Borowitz M.J., Le Beau M.M., Bloomfield C.D., Cazzola M., Vardiman J.W. (2016). The 2016 revision to the World Health Organization classification of myeloid neoplasms and acute leukemia. Blood.

[8] Creutzig U., van den Heuvel-Eibrink M.M., Gibson B., Dworzak M.N., Adachi S., de Bont E., Harbott J., Hasle H., Johnston D., Kinoshita A. (2012). Diagnosis and management of acute myeloid leukemia in children and adolescents: Recommendations from an international expert panel. Blood.

[9] Im H.J. (2018). Current treatment for pediatric acute myeloid leukemia. Blood Res..

[10] Abrahamsson J., Forestier E., Heldrup J., Jahnukainen K., Jonsson O.G., Lausen B., Palle J., Zeller B., Hasle H. (2011). Response-guided induction therapy in pediatric acute myeloid leukemia with excellent remission rate. J. Clin. Oncol. Off. J. Am. Soc. Clin. Oncol..

[11] Moerloose B., Reedijk A., Bock G.H., Lammens T., Haas V., Denys B., Dedeken L., Den Heuvel-Eibrink M.M., Te Loo M., Uyttebroeck A. (2019). Response-guided chemotherapy for pediatric acute myeloid leukemia without hematopoietic stem cell transplantation in first complete remission: Results from protocol DB AML-01. Pediatr. Blood Cancer.

[12] Locatelli F., Masetti R., Rondelli R., Zecca M., Fagioli F., Rovelli A., Messina C., Lanino E., Bertaina A., Favre C. (2015). Outcome of children with high-risk acute myeloid leukemia given autologous or allogeneic hematopoietic cell transplantation in the aieop AML-2002/01 study. Bone Marrow Transpl..

[13] Pession A., Masetti R., Rizzari C., Putti M.C., Casale F., Fagioli F., Luciani M., Lo Nigro L., Menna G., Micalizzi C. (2013). Results of the AIEOP AML 2002/01 multicenter prospective trial for the treatment of children with acute myeloid leukemia. Blood.

[14] Shim Y.J., Lee J.M., Kim H.S., Jung N., Lim Y.T., Yang E.J., Hah J.O., Lee Y.H., Chueh H.W., Lim J.Y. (2018). Comparison of survival outcome between donor types or stem cell sources for childhood acute myeloid leukemia after allogenic hematopoietic stem cell transplantation: A multicenter retrospective study of Study Alliance of Yeungnam Pediatric Hematology-oncology. Pediatr. Transpl..

[15] Mo X.D., Zhang X.H., Xu L.P., Wang Y., Yan C.H., Chen H., Chen Y.H., Han W., Wang F.R., Wang J.Z. (2016). Unmanipulated Haploidentical Hematopoietic Stem Cell Transplantation in First Complete Remission Can Abrogate the Poor Outcomes of Children with Acute Myeloid Leukemia Resistant to the First Course of Induction Chemotherapy. Biol. Blood Marrow Transplant. J. Am. Soc. Blood Marrow Transpl..

[16] Xue Y.J., Cheng Y.F., Lu A.D., Wang Y., Zuo Y.X., Yan C.H., Suo P., Zhang L.P., Huang X.J. (2020). Efficacy of Haploidentical Hematopoietic Stem Cell Transplantation Compared With Chemotherapy as Postremission Treatment of Children With Intermediate-risk Acute Myeloid Leukemia in First Complete Remission. Clin. Lymphoma Myeloma Leuk..

[17] Lazzarotto D., Candoni A., Filì C., Forghieri F., Pagano L., Busca A., Spinosa G., Zannier M.E., Simeone E., Isola M. (2017). Clinical outcome of myeloid sarcoma in adult patients and effect of allogeneic stem cell transplantation. Results from a multicenter survey. Leuk. Res..

[18] Zhou T., Bloomquist M.S., Ferguson L.S., Reuther J., Marcogliese A.N., Elghetany M.T., Roy A., Rao P.H., Lopez-Terrada D.H., Redell M.S. (2020). Pediatric myeloid sarcoma: A single institution clinicopathologic and molecular analysis. Pediatr. Hematol. Oncol..

[19] Kawamoto K., Miyoshi H., Yoshida N., Takizawa J., Sone H., Ohshima K. (2016). Clinicopathological, Cytogenetic, and Prognostic Analysis of 131 Myeloid Sarcoma Patients. Am. J. Surg. Pathol..

[20] Dusenbery K.E., Howells W.B., Arthur D.C., Alonzo T., Lee J.W., Kobrinsky N., Barnard D.R., Wells R.J., Buckley J.D., Lange B.J. (2003). Extramedullary leukemia in children with newly diagnosed acute myeloid leukemia: A report from the Children’s Cancer Group. J. Pediatric Hematol. Oncol..

[21] Gibson B.E.S., Webb D.K.H., Howman A.J., De Graaf S.S.N., Harrison C.J., Wheatley K. (2011). Results of a randomized trial in children with Acute Myeloid Leukaemia: Medical Research Council AML12 trial. Br. J. Haematol..

[22] Wennström L., Edslev P.W., Abrahamsson J., Nørgaard J.M., Fløisand Y., Forestier E., Gustafsson G., Heldrup J., Hovi L., Jahnukainen K. (2016). Acute Myeloid Leukemia in Adolescents and Young Adults Treated in Pediatric and Adult Departments in the Nordic Countries. Pediatr. Blood Cancer..

